# Screening of Mineralogenic and Osteogenic Compounds in Zebrafish—Tools to Improve Assay Throughput and Data Accuracy

**DOI:** 10.3390/ph15080983

**Published:** 2022-08-10

**Authors:** Joana T. Rosa, Marco Tarasco, Paulo J. Gavaia, M. Leonor Cancela, Vincent Laizé

**Affiliations:** 1 Centre of Marine Sciences, University of Algarve, 8005-139 Faro, Portugal; 2 S2AQUA—Collaborative Laboratory, Association for a Sustainable and Smart Aquaculture, 8700-194 Olhão, Portugal; 3 Faculty of Medicine and Biomedical Sciences, University of Algarve, 8005-139 Faro, Portugal; 4 GreenColab—Associação Oceano Verde, University of Algarve, 8005-139 Faro, Portugal; 5 Algarve Biomedical Center, University of Algarve, 8005-139 Faro, Portugal

**Keywords:** drug discovery, screening pipeline, bone anabolic compounds, zebrafish *Danio rerio*, technological innovation, high throughput

## Abstract

Bone disorders affect millions of people worldwide and treatments currently available often produce undesirable secondary effects or have limited efficacy. It is therefore of the utmost interest for patients to develop more efficient drugs with reduced off-target activities. In the long process of drug development, screening and preclinical validation have recently gained momentum with the increased use of zebrafish as a model organism to study pathological processes related to human bone disorders, and the development of zebrafish high-throughput screening assays to identify bone anabolic compounds. In this review, we provided a comprehensive overview of the literature on zebrafish bone-related assays and evaluated their performance towards an integration into screening pipelines for the discovery of mineralogenic/osteogenic compounds. Tools available to standardize fish housing and feeding procedures, synchronize embryo production, and automatize specimen sorting and image acquisition/analysis toward faster and more accurate screening outputs were also presented.

## 1. Introduction

Human disorders characterized by a progressive loss of bone mineral density (e.g., osteopenia and osteoporosis) or a gradual deterioration of bone architecture and deformation of bone shape (e.g., Paget’s disease of bone) affect a large number of people worldwide, resulting in reduced patient well-being and imposing a large financial burden upon society [1,2,3]. Several drugs acting on bone resorption or formation are available to treat these bone disorders and minimize their impact, but most of them have issues related to efficacy and/or undesirable secondary effects [1,2,3,4,5]. It is therefore of the utmost interest to develop more efficient drugs with reduced off-target activities.

In the long process of drug development, screening and preclinical validation are commonly performed using rodents (mostly mouse) as they are evolutionarily close to humans [6]. However, because of several bottlenecks related to rodents being an expensive and time-consuming animal model [7,8], but also to the relative low throughput of rodent-based in vivo screening systems [9,10], alternative systems are sought to reduce the number of compounds to be tested in rodents and thus accelerate drug discovery. In vitro assays using mammalian osteoblast, osteoclast, and bone marrow-derived mesenchymal stem cell cultures have been established for high-throughput screening assays toward the discovery of osteogenic compounds [11,12,13,14,15], but they lack the cellular complexity of the in vivo models.

The quest for systems that could bridge the gap between simple high-throughput cell-based assays and complex low-throughput whole-animal assays identified small aquatic vertebrates as promising tools for the screening of compounds [16], particularly those that potentially have an effect on bone metabolism. The zebrafish, a small teleost endogenous to the streams of the Southern Asia, brings many advantages over the mammalian models. Because of its short life cycle, large progeny, see-through larval bodies, and low maintenance costs, but also because it shares with mammals a remarkable homology of the mechanisms regulating bone development and homeostasis [17,18], it has rapidly gained momentum as a vertebrate model organism to study pathological processes related to human bone disorders [19,20,21], and to screen for bone anabolic compounds [19,22,23,24,25]. Given its small size and cost-effective husbandry, the zebrafish also has the capacity to downscale whole-organism screening platforms and reduce associated costs, thus increasing the throughput of the screening pipeline, and accelerating the identification of compounds with therapeutic applications. CRISPR/Cas9 gene editing technology has also been successfully applied to zebrafish [26,27], facilitating the development of gene-specific mutant lines exhibiting phenotypes/traits of bone disorders (e.g., osteoporosis, osteopenia, and osteoarthritis) that will allow more efficient and better targeted drug screening efforts. However, despite clear advantages, translational validation, and availability of robust zebrafish models, the skepticism of the pharmaceutical industry has hindered a wider use of zebrafish in screening pipelines for drug discovery.

The first approaches to screen for osteoactive compounds were largely empirical but several in vitro (e.g., mineralogenic cell lines), ex vivo (e.g., scale cultures), and in vivo (e.g., embryonic and larval bone structures) systems have now been developed and recently optimized (see description below). The assessment of the mineralogenic/osteogenic potential of selected compounds is mostly based on the morphometric analysis of bone structures stained with bone-specific dyes (e.g., alizarin red S and calcein) or marked with reporter proteins (e.g., GFP and mCherry) expressed by transgenic zebrafish lines (see description below). The *modus operandi*—including turnaround time, system throughput, operating skills and requirements in compounds—and the cellular complexity greatly varies among the different systems available but a major bottleneck common to all systems is probably the extensive time required to image the bone structures and assess their morphometry (e.g., size and shape) and density (e.g., pixel intensity) through image analysis. Key processes have recently been optimized and automated, procedures in animal production have been progressively standardized, and sophisticated tools have been developed to increase the throughput and the accuracy of the zebrafish screening systems. This review presents the zebrafish systems that can be used to screen for mineralogenic/osteogenic compounds and highlights the tools available to increase their throughput for faster and more accurate preclinical studies.

## 2. Zebrafish In Vitro and In Vivo Screening Systems

### 2.1. In Vitro Cell Systems Capable of Biomineralization

**Figure 1:**
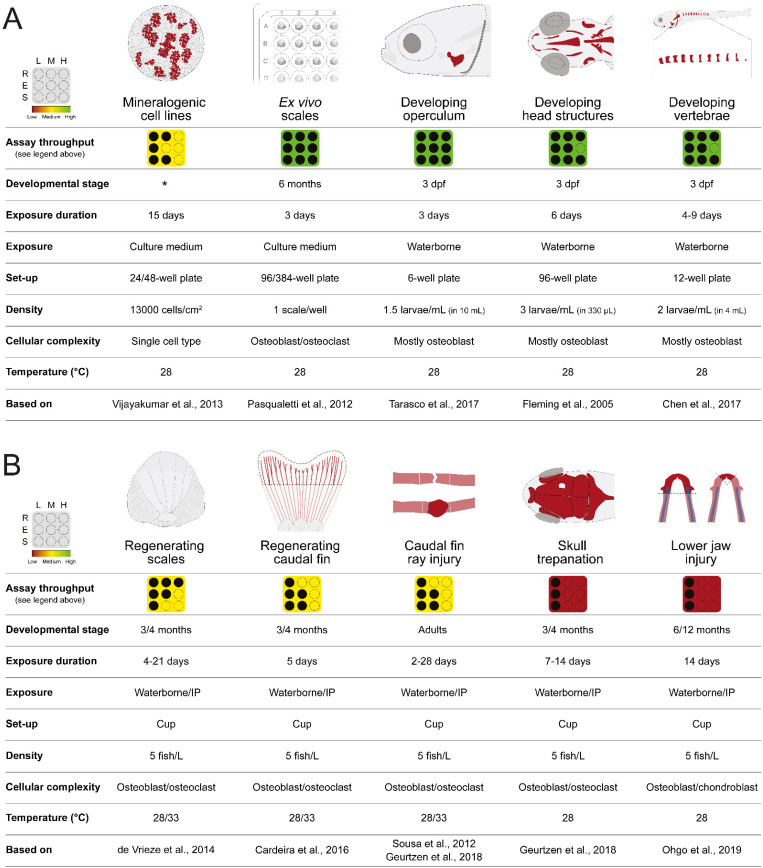
Overview of the zebrafish assays currently available for the screening of osteoactive extracts or compounds. (**A**) Zebrafish models of bone development [22,23,30,42,51]. (**B**) Zebrafish models of bone repair and regeneration. R, number of experimental units; E, exposure duration; S, set-up; L, low; M, medium; H, high; dpf, days post-fertilization; IP, intraperitoneal injection; * Cell line developed from a mixture of calcified tissues from juvenile zebrafish and composed of osteoprogenitor cells [55,58,70,72,73].

Many cell lines of fish origin are available (856 in the Cellosaurus version 42 of June 2022 [28]) to study intracellular signaling pathways, gene transcriptional regulation and metabolic functions with application in biomedical, aquaculture, and environmental studies. However, only 11 of these fish cell lines are suitable to assess compound mineralogenic potential, i.e., the capacity to enhance (pro-mineralogenic) or reduce (anti-mineralogenic) extracellular matrix (ECM) mineralization (see Laizé et al. (2022) for a list of the fish cell lines capable of in vitro mineralization [29]). ZFB1 (Cellosaurus accession no. CVCL_6E12) is the only zebrafish cell line capable of in vitro mineralization [30]. While it has been used to investigate the expression of several bone-related genes throughout in vitro mineralization [30,31], it has not yet been employed in screenings for osteogenic compounds. On the contrary, gilthead seabream VSa13 cell line ([32]; Cellosaurus accession no. CVCL_S952) was successfully used in small-scale screening of mineralogenic extracts from marine green algae [33], halophytes [34], cyanobacteria (J. Rosa unpublished data), or environmental osteotoxic pollutants [35,36]. Typically, compounds are dissolved in culture medium and exposed to the mineralizing cells in multiwell plates (Figure 1A). Culture medium supplemented with both the compounds and mineralogenic cocktail—ascorbic acid, calcium chloride and β-glycerophosphate—is renewed twice a week for 14 days, then the extent of in vitro mineralization is assessed through alizarin red S (AR-S) staining and quantified by spectrophotometry [37]. In addition to the low quantity of compounds needed and the cost-effective method to induce ECM mineralization, fish mineralogenic cell lines are also advantageous as an in vitro cell system for drug screening because of their robustness and high mineralogenic capacity. A rather long exposure is currently the major bottleneck in the use of cell lines to screen for mineralogenic compounds. However, there is room for optimization: a stable cell clone expressing a fluorescent protein under the control of a mineralization-specific gene promoter (see [38] for genes up-regulated during ECM mineralization) could be used as a proxy for cell mineralogenic capacity and a reporter for compound mineralogenic activity. Such a system would reduce the exposure time and remove the staining step, and consequently enhance screening throughput.

### 2.2. Bone Formation in Larval Systems

The use of zebrafish embryos and larvae for compound screening and drug discovery brings several advantages in relation to throughput and ethics. Zebrafish embryo and larvae are small (approximately 0.7 mm for a fertilized egg and 4.2 mm for a larva at 6 days post-fertilization (dpf); Zfin) and can be easily handled using a wide bore transfer pipette or a robotic handling system (see Section 3). They can be arrayed in multiwell plates (commonly 96-well plates) in a small volume of water, hence requiring only small amounts of compounds for screening assays. Because zebrafish larvae can feed on their yolk reserves for several days, external feeding is not required until 6 dpf, which is the maximum time to host zebrafish larvae in 96-well plates. For all these reasons, the use of zebrafish embryos and larvae for high-throughput screening should be limited to a developmental window that ranges from fertilized eggs to 6 dpf larvae. Assays using zebrafish larvae older than 120 h post-fertilization (5 dpf) require approval from an ethical committee [39], a limitation that may reduce the developmental window for the screening of some compounds. Anyhow, the exposure of zebrafish embryo/larvae to compounds is likely to be short, therefore enhancing screening throughput and reducing the amount of each compound to be tested. As described in this review, several dermal bone structures are being formed at 3 dpf and are sufficiently mineralized at 6 dpf to be used for the screening of osteogenic compounds, e.g., parasphenoid, branchiostegal rays, and operculum [40,41]. Using older larvae, e.g., to assess bone structures that are being formed and/or mineralized later (for example vertebral centra) will require feeding, transfer to larger wells or tanks, and use of larger amounts of compounds, and will thus decrease the throughput of the screening pipeline.

#### 2.2.1. Opercular Bone Growth

A dermal bone with flat morphology and a superficial localization on the side of the head, combined with an early and rapid ossification in larval zebrafish [42], are key features that accelerated the use of zebrafish operculum to study osteoactive [33,43,44,45] and osteotoxic compounds [35,42,46], and spot it as a promising system for large-scale compound screenings. Briefly, 3 dpf larvae are exposed to compounds of interest for 3 days in multiwell plates, and then bone structures are stained with AR-S or calcein and imaged for morphometric analysis of the operculum area (Figure 1A). To correct for inter-specimen size variability, the area of the head is used to normalize the area of the operculum. Increase or decrease in corrected operculum area indicates compounds with pro- or anti-osteogenic (or osteotoxic) properties, respectively [42].

The operculum assay is fast (4 days from exposure to data acquisition) and requires a low quantity of molecules (exposure is short and done in a reduced volume of fish water), but also few resources that are commonly found in most research institutes or easily implemented (mainly a thermostatted chamber or incubator, a fluorescence stereomicroscope, and an image analysis software). It is also adaptable to different experimental settings (specimen number, well plates, number of conditions, and treatment volume) and it is performed with early-stage larvae that can be produced in large quantities. ImageJ macros have been developed to speed up the rather laborious morphometric analysis [47] and tools are available to automatize larvae and liquid handling but also operculum imaging (see Section 3). Note that the larval operculum is a simple bone structure containing mostly osteoblasts at this stage (6 dpf). This could be seen as an advantage to discover bone anabolic compounds, but the absence of osteoclasts at this early developmental stage is a clear drawback if screening aims at anti-resorptive molecules.

#### 2.2.2. Craniofacial Skeleton

Zebrafish craniofacial bones are ossified following mechanisms that have been conserved throughout vertebrate evolution and are therefore commonly used as models for mammalian cranium development [48]. Fleming et al. investigated the osteogenic potential of several molecules by assessing the mineralization of several craniofacial bones in zebrafish larvae [22]. At 3 dpf, larvae were exposed for 6 days in multiwell plates to osteoactive compounds, then stained with AR-S and area and staining density of ventral cranial bone structures was evaluated (Figure 1A). The overall procedure is similar to that used in the operculum assay although exposure is longer, thus requiring more compounds, and mineralization data are not normalized for inter-specimen variability, therefore necessitating a higher number of specimens to reach statistical significance. The morphometric analysis of multiple bone structures may provide more robust data but will also increase the time of data analysis, which is an issue for large scale screenings. As for the operculum assay, laborious steps such as image acquisition and analysis but also specimen handling can be automated to provide a higher throughput for drug discovery pipelines.

#### 2.2.3. Vertebrae Mineralization

Zebrafish vertebral centrum is formed throughout intramembranous ossification that can be detected as early as 7 dpf, when part of the notochord sheath began to mineralize [49]. Quantification of vertebrae mineralization—the number of mineralized vertebral centra or the extent of centrum mineralization—can be used as a tool to evaluate the mineralogenic or osteogenic activity of selected compounds or molecule libraries, as reported by Chen and colleagues for dorsomorphin, pentamidine, fenvalerate, and alendronate, among others [23]. Zebrafish larvae are typically exposed to selected compounds/libraries for 4–9 days then stained with AR-S or calcein at 7–10 dpf and imaged for analysis (Figure 1A). As for the other larval systems, the advantages of this system are the low amount of material needed and the fast read out, while image analysis remains a major bottleneck. The automation of specimen handling and image acquisition/analysis is available to reduce procedure time and increase screening throughput.

### 2.3. Ex Vivo Culture of Elasmoid Scales

Zebrafish elasmoid scales are small bone-like units of easy access that can be easily and rapidly plucked out of zebrafish skin. They are available in rather large quantities—around 200 elasmoid scales in a single adult zebrafish [50,51]—and can be cultured ex vivo for several days [52,53]. Thus they have been successfully used to study and discover osteogenic drugs [54,55] (Figure 1A). Scales are usually removed from the flank of the adult zebrafish in the region from behind the head to the anterior margin of the anal fin [56]. To ensure minimal damage and fish survival, only a limited number of scales should be plucked out. Although this has not been tested thoroughly, approximately 50 scales can be removed without a significant increase in mortality [56,57]. Upon removal, scales regenerate and can be used to assess the potential of test compounds to affect de novo bone formation (see next section). Simple staining procedures—e.g., TRAP (tartrate resistant acid phosphatase) staining to assess osteoclast bone resorbing activity and ALP (alkaline phosphatase) staining to assess osteoblast bone forming activity, but also von Kossa’s staining to assess scale mineralization and patterning—have boosted the interest of this system for high-throughput screening. The availability of transgenic lines for bone marker genes, e.g., *Tg(sp7:mCherry)* or *Tg(Ola.Sp7:Luciferase)*, where an easily quantifiable reporter signal is used as a proxy for bone formation, has accelerated the screening procedure by shortening ex vivo culture, limiting scale handling and simplifying image acquisition [54]. The small size of adult zebrafish scales, less than 1 mm, is also a clear advantage for screening pipelines as they can be individualized in 384-well plates, therefore decreasing the quantity of compounds needed and enhancing throughput. While most steps of the screening pipeline using ex vivo cultures of elasmoid scales—including scale sorting in multiwell plates—can be automated to further enhance throughput, the plucking of the scales has still to be performed manually as it requires precision and care to avoid scale damages or animal injuries. It is also worth mention that not all scales have the same size or the same morphology [56], justifying the need to normalize data (e.g., fluorescence signal or bone-specific staining) to correct for inter-scale variability.

### 2.4. Bone Structures Capable of Repair and Regeneration

**Figure 2:**
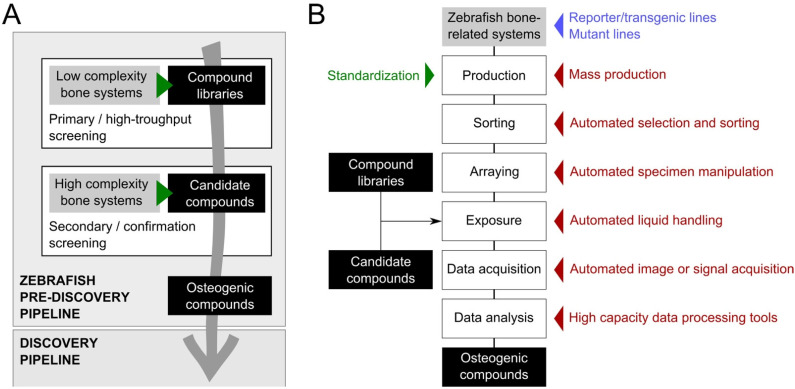
Zebrafish pre-discovery pipeline (**A**) and technological innovation applied to the standardization and automation to improve zebrafish screening throughput and data accuracy (**B**).

The ability of the zebrafish to fully restore damaged/amputated skeletal structures—e.g., fin rays, skull, jaw and scales—has been used to study molecular and cellular mechanisms underlying de novo bone formation [58,59,60,61,62,63,64]. Upon surgical amputation of the caudal fin, plucking of scales, craniectomy, or injuries to fin rays or the lower jaw, a regenerative program initiates and missing or damaged tissues, including bone and bone-like structures, are rapidly and faithfully restored or repaired. Assays aiming at assessing bone repair and regeneration typically involve adult zebrafish, which are much lower throughput that embryos and larvae (e.g., adult specimens require larger housing facilities, more time to reach experimental size, and bigger handling equipment). Exposure to test compounds is also commonly carried out by immersion, which in the case of adult specimens requires a larger volume of water (each adult zebrafish is kept in 250 mL of fish water), and thus a larger amount of the compounds. For these reasons, it is safe to say that these systems should be limited to secondary screening, where the osteogenic potential of promising compounds identified in primary high-throughput screenings are further confirmed and characterized for underlying mechanisms (Figure 2A). Nonetheless, a handful of procedures and tools can be implemented to reduce the quantity of compounds used to expose adult zebrafish. For example, intraperitoneal injection is a procedure that can be safely applied to adult zebrafish to deliver a small quantity of compound into the abdominal cavity, posterior to the pelvic girdle [65]. It can be easily implemented in any laboratory; however, it is not yet automated and should therefore be limited to compounds available in very low quantity or insoluble in water. If waterborne exposure remains preferable, ScreenCube can be used; it is a 3D printed housing system for intermittent drug dosing that allow a 10-fold reduction in the quantity of compounds needed to expose adult zebrafish [66].

#### 2.4.1. Regenerating Caudal Fin

Zebrafish caudal fin is a simple structure composed of bony rays spaced with vascularized and innervated connective tissue and covered with a pigmented epidermis [67]. Upon amputation of the fin (i.e., finectomy), tissues are restored through epimorphic regeneration in less than two weeks [68]. The simple structure and the remarkable regenerative properties of zebrafish caudal fin have fostered its use in screening assays aiming at identifying compounds, or extracts, with bone anabolic or mineralogenic effects [35,44,58,69] (Figure 1B). Cardeira et al. [58] and Tarasco et al. [47] have recently optimized the experimental procedures to reduce assay duration: tissue restoration is accelerated by placing finectomized zebrafish at 33 °C (instead of 28 °C), regenerative and osteogenic potential of selected compounds are assessed at 5 days post-amputation, and morphometric analysis of the regenerated fin is semi-automatized using ZFBONE ImageJ macro. Advantages of the caudal fin regeneration assay are related to its capacity to provide data at multiple levels, i.e., bone regeneration by determining the extent of de novo bone formation, bone mineralization by quantifying bone mineral density, and bone patterning by assessing the bifurcation of fin rays. Beside the bottlenecks related to the use of adult fish (see above), a major limitation of the regenerating caudal fin system that can affect screening throughput is the finectomy—a surgery necessitating fish manipulation and anesthesia, and performed under a stereomicroscope, thus a rather laborious procedure—that can hardly be automated.

#### 2.4.2. Regenerating Elasmoid Scales

When they are lost or removed experimentally, zebrafish elasmoid scales can fully regenerate within 2–3 weeks following a process comparable to intramembranous ossification [64]. As for the regenerating caudal fin rays, regenerating scales can be used to assess or study compound regenerative and osteogenic potential (Figure 1B). In this experimental setup, fish have to survive scale removal while regeneration proceeds, and hence only 20–30 scales should be removed from the fish flank, just prior to fish being exposed to compounds for 4 to 21 days (typically 5 days) until regenerated scales are collected and analyzed. As for the regeneration of the caudal fin, increasing water temperature to 33 °C will accelerate the regeneration of the elasmoid scales, which can be used at 5 days post-plucking to screen for molecules with osteogenic potential. Morphometric analysis of regenerated scales can provide information on scale area, morphology, osteoclast activity and mineral deposition to gain insights on the bone remodeling process. Besides the bottlenecks related to the use of adult fish (see above), a major limitation of the regenerating scale system that can affect screening throughput is the plucking—a procedure requiring fish manipulation and anesthesia, and performed under a stereomicroscope, thus a rather laborious procedure—that can hardly be automated.

#### 2.4.3. Bone Repair

Injuries to bony rays and cranial bones in the adult zebrafish have been used to model human bone fractures and study bone repair [70,71,72]. In the first assay, bony rays of 3–6 months old adult zebrafish are crushed using forceps (typically in the middle of a segment anterior to bifurcation), and bone repair is monitored upon exposure to selected compounds (Figure 1B). Ossification initiates at the fracture sites at about 3 days post-injury (dpi) and a bone callus is visible from 5–6 dpi until 28 dpi (or longer), although it becomes thinner in response to active remodeling at the fracture site. Typically, bone formation is assessed by AR-S staining at 6–11 dpi and the effects of the compounds determined through the morphometric analysis of fluorescence images. Although this has not been tested thoroughly, several rays can be injured in the same fish (up to 4, in non-adjacent rays and in the 2 fin lobes [71]) to maximize data collection and compound usage. In the skull repair assay, injuries of about 0.5 mm are inflicted to the frontal or parietal bones of the cranial vault (homologous to bones of the mammalian neurocranium) in anesthetized adult zebrafish, using a microdrill [72]. Most of the healing process occurs between 3 and 14 dpi. During this period and upon the administration of selected molecules, de novo bone formation can be monitored and imaged to assess osteogenic effects. To the best of our knowledge, bone crush, skull injury, and associated morphometrics are not yet automated procedures, but other procedures such as fish and compound handling, and image acquisition can be accelerated by using the tools presented in Section 3.

#### 2.4.4. Regenerating Lower Jaw

The lower jaw of adult zebrafish is a simple skeletal structure primarily composed of the mandibular bone and Meckel’s cartilage [73]. Upon amputation, a cartilaginous structure is formed and later surrounded by the new bone. In the case of a proximal amputation (both mandibular bone and Meckel’s cartilage are present at the amputation plane), cartilage will remain after the complete regeneration of the lower jaw, while it will disappear in the case of a distal amputation (only mandibular bone is present at the amputation plane) (Figure 1B). The use of adult fish (thus the need for larger amounts of compounds), and the long regeneration time > 30 days (thus the need for longer exposure time) are probably the major bottlenecks of this assay. In addition, the surgical removal of the jaw fragment upon anesthesia and the morphometric analysis of the regenerated structure are two laborious procedures that are not yet automated, i.e., no robotic solution for the surgery and no macro for image analysis are available. As for the regenerating caudal fin system, the regenerating lower jaw system should be limited to secondary screening, where the osteogenic potential of promising compounds identified in primary high-throughput screenings are further confirmed and characterized for underlying mechanisms (Figure 2A).

### 2.5. Zebrafish Lines for Phenotypic Screening of Bone Anabolic Compounds

Molecular mechanisms and cell types associated with skeleton development and mineralization have been remarkably conserved throughout evolution [19,20,74,75,76] and 80% of human disease-related genes have well-conserved orthologs in the zebrafish genome [77]. This, together with the amenability of zebrafish genome to genetic manipulation, prompted the development of genetic and transgenic variants to study human skeletal genetic disorders in basic and pre-clinical research. Gene editing techniques used to generate zebrafish skeletal disease models have evolved rapidly. At first, random point mutations induced by chemicals mutagens (e.g., 1-ethyl-1-nitrosourea (ENU) [78]) were used to generate mutant lines with bone phenotypes that resemble human disorders (e.g., the *chihuahua* mutation in *col1a1* to mimic osteogenesis imperfecta [79]). The development of zebrafish mutant lines recently gained momentum with major advances in genome sequencing [77] and genome editing tools, e.g., TALEN [80] and CRISPR)/Cas9 systems [26,81]. These breakthroughs enabled the tailoring of gene-specific mutations (e.g., point mutations or deletion of specific gene regions) and the replication of the mutations identified through genome wide association studies (GWAS) and the sequencing of patient genomes [82,83,84,85]. Zebrafish is also a unique model to study the function of genes whose mutations in rodent orthologs are lethal. Indeed, teleost-specific whole genome duplication [86] resulted in the presence of two gene copies in zebrafish genome. In many cases, the two copies were maintained so that a viable loss of function can be obtained and studied due to a partitioning of gene function [87].

Several excellent reviews addressing the use of zebrafish mutant lines to study skeletal disorders have been published [19,20,74,75,88,89,90,91], whose content will not be repeated here. According to the most recent review, more than 80 different zebrafish mutant lines are available to study 78 human skeletal disorders [90], out of the 461 classified by the Nosology Committee of the International Skeletal Dysplasia Society [92]. Additionally, approximately 50 transgenic reporter lines have been developed and used to investigate zebrafish skeleton (see refs. [20,88] for a list of these transgenic lines). Many of these mutant and transgenic lines are accessible through repositories such as the Zebrafish International Resource Center (ZIRC) and the European Zebrafish Resource Center (EZRC), while important data—e.g., genes, alleles, transgenic lines, gene expression profiles, phenotypes and orthology—are available at the Zebrafish Information Network (ZFIN).

Besides the lines mentioned above—those developed to model skeletal disorders and assess the expression of skeletal marker genes—other lines have sparked some interest because of the technical advantages they could bring to the study and screening of bone anabolic and catabolic compounds. In this regard, *casper* [93] and *crystal* [94] mutations generate a ghost-like phenotype that allow juvenile and adult stages to have a translucent body, a feature limited to embryonic and larval stages in other zebrafish lines. When this see-through attribute is associated with a fluorescent marker (e.g., green, red, yellow or blue fluorescent proteins) or a fluorescent stain specific for mineralized tissues (e.g., AR-S and calcein), it allows real-time live imaging. This can be used to follow the progression of skeletal pathologies in the different mutant lines but also to screen for molecules capable of rescuing disease phenotypes, study their mechanisms of action and possible toxic and side effects in the different organs.

Exposure of zebrafish—mutant, transgenic or wild-type lines—to an excess of glucocorticoids, iron or dietary fat can also be used to mimic human bone metabolic disorders such as osteoporosis [25]. Osteoporotic fish can then be used in screening pipelines aiming at discovering novel drugs with the ability to rescue or prevent disease phenotype. It is worth mention that many drugs active in humans trigger similar effects in zebrafish, and this is particularly true for osteogenic compounds such as calcitriol, parathyroid hormone (PTH), bisphosphonates, and several natural compounds (reviewed in ref. [25]).

## 3. Tools to Improve Screening Throughput and Replicability

Technological innovation applied to the standardization and mass production of the animals, to the robotic handling of animals, plates, and liquids, as well as to the automation of data acquisition and processing, can improve screening throughput and data accuracy, thus accelerating the use of zebrafish systems for bone anabolic/catabolic drug discovery (Figure 2B). The miniaturization and improvement of various pieces of apparatus have also boosted screening capacity by reducing operational times and the need for biological material. However, technological innovation has a cost: it is expensive to set up and to run and therefore requires a massive investment that many research laboratories in academia or small companies cannot afford. A larger implementation of high-throughput zebrafish screening pipelines will only be possible if costs associated with the technologies that accelerate screening speed markedly decrease in the near future.

### 3.1. Inbred Zebrafish Lines

Wild-type zebrafish lines found in pet shops worldwide exhibit a relatively high degree of genetic diversity that triggers some variability in their phenotype, and consequently some variability in their response to compound exposure [95]. In this regard, several inbred laboratory lines have been developed (e.g., AB and TU lines; see ZFIN for a comprehensive list of wild-type laboratory lines) to reduce genetic diversity and response variability; these strains should be preferentially used in drug discovery pipelines as they will increase data accuracy and therefore reduce the need for higher number of replicate experiments.

### 3.2. Standardized Housing and Feeding

Animals for screening purposes must be produced under conditions that ensure optimal growth and welfare to provide homogeneous and replicable data. The standards of an ‘optimal growth and welfare’ may vary considerably between wild-type and mutant lines—it largely depends on the severity of the mutant phenotype—and the term ‘most favorable growth and welfare’ is probably more appropriate. To provide optimal/most favorable conditions, housing, husbandry and feeding of the zebrafish must be adapted to all life stages and standardized. The European community directive 2010/63/EU on the protection of animals used for scientific purposes establishes general guidelines for water quality, chemical and physical parameters applicable to all fish species; however, it does not specify zebrafish requirements.

Recommendations for housing and husbandry of zebrafish were recently refined (e.g., Aleström and colleagues in 2020 [39]), providing a comprehensive guide on the best practices for rearing and maintaining zebrafish under optimal growth and welfare conditions, emphasizing that standardization of husbandry procedures is essential for improving experimental replicability, and providing acceptable ranges for different parameters (see Table 1 for a summary). Fish density is a critical parameter to achieve optimal growth and welfare conditions. Although stocking densities of 4–10 adults/L and 25 juveniles/L are commonly used [39,96], lower densities are recommended to keep oxygen levels close to saturation, maximize growth rate, and reduce stress, thus increasing welfare [97]. High fish density also increased the proportion of males in the breeding stock [98] and decreased the production of eggs [99]. It is therefore important to maintain optimal housing conditions for zebrafish used in screening experiments but also for breeders used to produce high-quality eggs.

The nutrition of model animals has been recognized as an important variable in research outcomes and the use of ‘standard reference diets’ has been proposed to improve the replicability of scientific data [104]. Among the several zebrafish facilities dedicated to research worldwide, variations in feeding protocols that could affect growth, health, and behavior have been identified; the use of live preys with different nutritional value and the introduction of dry food at different developmental stages are striking examples [26,97,100,101,105,106,107,108,109,110]. Efforts have been made recently to uniformize zebrafish feeding procedures but also to use standard diets with controlled nutritional composition [111]. In this regard, two microdiets have been commercialized under the commercial names ZEBRAFEED (Sparos Lda) and GEMMA Micro (Skretting); they are formulated to meet the nutritional requirements for all life stages of zebrafish, therefore removing the need for live prey (Table 2). While both diets provided optimal growth conditions, ZEBRAFEED promoted a higher embryo and larval survival and enhanced reproductive performance [109]. The large-scale usage of a microdiet following an established feeding protocol will not only contribute towards the standardization of zebrafish husbandry and the replicability of research data but also maximize the production and quality of zebrafish embryo/larvae for drug or mutagenesis screenings [109]. In the context of this review, the implementation of a standard reference diet optimized for nutrients essential to the correct development of fish skeletal structures—e.g., fatty acids, phospholipids, vitamins, and minerals—will decrease the incidence of skeletal deformities [111,112], providing better conditions for the screening of osteogenic compounds. A ‘challenging’ diet that would increase the incidence of skeletal deformities may be used if the objective of the screening is to identify compounds that can improve skeletal status by reducing the rate of deformities. In this regard, Sparos Lda (www.sparos.pt, accessed on 22 May 2022) produces tailor-made diets for zebrafish.

### 3.3. Mass Production of Synchronized Embryos

To generate statistically meaningful results, zebrafish high-throughput screening requires the daily production of thousands of synchronized embryos (i.e., at the same developmental stage). To decrease the intensive manual labor that would result from the manipulation of dozens of tanks and hundreds of breeders and increase embryo synchronization, large-scale egg production systems have been developed [117]. Commercial breeding systems such as the Mass Embryo Production System (MEPS; Aquatic Habitats) and iSpawn (Tecniplast) can achieve remarkable spawning rates of up to 800–1000 embryos/minute (Table 2). More affordable and with a good spawning capacity (e.g., 10,000 embryos per breeding session using 100–200 pairs of fish), custom-made mass embryo production systems are also available [114]. To maintain an optimal mass production of embryo—both qualitatively and quantitatively—breeders that may suffer some stress incurred by frequent group mattings should be replaced regularly, e.g., using a rotation of 1000 breeders per week.

### 3.4. Target Specimen Sorting

Targets for drug screening in zebrafish can be cells, tissues or whole animals (embryo and larvae). While cells—adherent or in suspension—can be easily arrayed in multiwell plates using liquid-handling robots, tissues and whole animals have been typically sorted out under a stereomicroscope using wide-bore transfer pipettes, in a rather laborious process. Automation of this process can be carried out using a fluorescence-gated sorting system—e.g., the COPAS FP-1000/2000 of Union Biometrica (Table 2)—that has the capacity to array tissues, embryos and larvae in 96- and 384-well plates but also select them based on viability (dead embryo are discarded), morphology (e.g., size and length) and/or fluorescent signal, and sort a desired number of specimens into individual wells of a microtiter plate. Custom-made systems have also been developed to automatically sort embryos into microplates, e.g., the ZebraFactor of the Swiss Center for Electronics and Microtechnology [113], but also to automatically remove the chorion (a barrier that may prevent or limit embryo exposure to compounds) before sorting, when drug screening is performed in early stages of embryo development (prior hatching, which occurs between 48 and 72 hpf depending on rearing temperature) [118]. Another custom-made system has been developed to integrate embryo sorting, compound delivery, incubation, imaging, and image analysis in an automated high-throughput platform [119]. However, none of these custom-made systems have the ability to sort based on fluorescence or provide morphological information. In the context of this review, larvae from bone-specific transgenic lines expressing a fluorescent reporter protein or from wild-type lines stained with bone-specific fluorochromes can be easily sorted out using a fluorescence-gated sorting system. Typically, 4-dpf zebrafish larvae have cranial bone structures sufficiently mineralized to be detected using fluorochromes or have bone-related genes sufficiently expressed to be detected using reporters, thus are suitable for sorting [120]. Scales of transgenic fish—e.g., *Tg(sp7:mCherry)*—can also be efficiently sorted in 96- or 384-well plates based on a fluorescent signal, although they have to be first harvested manually from the fish as plucking has not yet been automated. In a general manner, automatic sorting is efficiently applied to tissues or whole animals in the range 0.1–10 mm.

### 3.5. Compound Delivery

Higher screening throughput and higher data accuracy can be achieved using liquid-handling robots to rapidly dilute and/or dispense precise amounts of the compounds from stock library plates and array targets. Robotic solutions are commercialized by different companies (e.g., PerkinElmer, Brand, Hudson Robotics, Labcyte, Gilson) and can be adapted to the zebrafish systems used to screen for osteogenic compounds. To further increase throughput screening, plate-handling robotic arms can also be used (e.g., PerkinElmer, Hudson Robotics), although their implementation requires a massive investment which probably limits their usage to big Pharma and Biotech companies. If compounds have to be delivered through microinjection, robotic injection systems have been developed: perivitelline injection for embryos [121,122] and intrayolk injection for larvae [123]. The microinjection robot commercialized by Life Science Methods (The Netherlands) is suitable for the injection of compounds into the chorion, yolk or first cell of zebrafish eggs at a rate of 1000 eggs in 25 min (Table 2).

### 3.6. Image Acquisition

To improve the throughput and quality of screening pipelines, image acquisition has to be automated and use rapid and high-resolution camera. In the case of zebrafish screening, the microscope objective should also have the capacity to capture large objects such as larvae. While examination of wild-type and mutant/diseased animals for developmental, morphological, and functional changes upon compound exposure can be done, the use of transgenic reporter fish expressing fluorescent proteins can greatly increase the automation of image acquisition. In this regard, fluorescent microscopes with automated stages (e.g., VAST BioImager from Union Biometrica, Imaging robot for small aquatic organisms from Life Science Methods, and High Content Screening LSI system from Leica) or microplate readers coupled to high resolution camera (e.g., ImageXpress system from Molecular Devices, EnSight Multimode Plate Reader from PerkinElmer and the Imaging Machine from Acquifer) will speed-up the examination and imaging (in the minute range) of zebrafish embryos or larvae (Table 2). Imaging systems are continuously being improved to achieve images with higher brightness and resolution, to bring three dimensionality, or to include densitometric and morphometric features (e.g., using high speed spinning disk confocal microscopy [124], confocal Raman microscopy [125], light sheet microscopy [126] and micro-computed tomography [127]). An automatically rotating capillary to orientate larval zebrafish, as in the VAST system, will also increase image quality and accelerate image acquisition.

### 3.7. Image Analysis

Automated screening systems generate large quantities of images that need to be analysed using specialized algorithms to provide accurate data in a timely manner. Commercial solutions and open-source software are available for automated image analysis, providing macros to count, measure, characterize morphometry, and classify objects. Most of them can also be used for image acquisition—providing tools to control microscope and capture images—and all of them can be easily applied to screening pipelines using zebrafish (Table 2; see also the review by Mikut et al. [128]). Of particular interest for this review, ZFBONE—a toolset gathering macros developed using ImageJ—is available to perform a semi-automatic morphometric analysis of several of the bone structures described in Figure 1 (e.g., operculum, scales and caudal fin rays) and increase the throughput of these assays [47].

## 4. Conclusions

Several assays recently developed in zebrafish to study mechanisms of bone development, repair, and regeneration, have the capacity to integrate high-throughput drug discovery pipelines if coupled with technological innovations applied to the standardization and automation of the screening process. However, to the best of our knowledge, a high-throughput screening pipeline using zebrafish for bone anabolic drug discovery has yet to be implemented. This may be related to the skepticism of the pharma/biotech industry about the validity of the zebrafish to model human diseases, in particular bone disorders. Technological features that can further increase replicability and throughput of zebrafish screening pipelines are developing rapidly and should bring an increased interest to this simple and cost-effective vertebrate model in relation to its capacity to achieve robust data comparable to those collected in rodents. In a different field of research, zebrafish is also gaining momentum as a model for acute/chronic toxicity studies of environmental pollutants (biocides, metals, microplastics, etc.) and drugs (safety assessment before marketing). Zebrafish assays and technological innovations described here can also be used to screen compounds for osteotoxicity [129].

## Figures and Tables

**Table 1:** Optimal parameters to standardize zebrafish housing conditions according to refs. [39,96,97,98,99,100,101,102,103].

Parameters		Description
Filters	Mechanical	Filter pads; cleaned daily and changed monthly
	Chemical	Activated charcoal; changed every 6 months
	Biological	Bio-balls or ceramic rings hosting nitrifying bacteria (*Nitrosomonas* and *Nitrobacter*)
Germicidal light		Ultraviolet light at 254 nm; bulbs changed after 6000 h of use
Temperature		24–29 °C (ideally 28.5 ± 0.5 °C)
Photoperiod		14 h of light|10 h of dark (automated light system to be checked regularly)
Water	Type	Dechlorinated water (ideally filtered reverse osmosis water)
	pH	6.5–8.0 adjusted with sodium bicarbonate
	Conductivity	150 to 1700 µS adjusted with commercial salts
	Hardness	3–8 d (ideally 4–5 d)
	Ammonia	< 0.1 mg/L (as close to 0 mg/L as possible)
	Nitrites	< 0.3 mg/L (as close to 0 mg/L as possible)
	Nitrates	<25 mg/L
	Renewal	5–10% in a daily basis (occasionally up to 15%)
Fish density		5 adults/L, 25 juveniles/L and 100 larvae/L

**Table 2:** Tools to improve the throughput and accuracy of in vivo screenings in zebrafish (all web pages accessed on 22 May 2022).

Tool (Company)	ZF Standardized Production	ZF Mass Production	ZF Sorting	Compound Handling	ZF Exposure	ZF Handling	Signal Acquisition	Imaging	Data Analysis	URL/Reference *
ZEBRAFEED (*Sparos Lda.*)	X									www.sparos.pt
GemmaMicro (*Skretting*)	X									www.skretting.com
MEPS—Mass embryo production systems (*Aquatic Habitats*)		X								www.mbki.com
iSPAWN (*Tecniplast*)		X								www.tecniplast.it
COPAS FP-1000/2000 (*Union Biometrica*)			X							www.unionbio.com
ZebraFactor (*Swiss Center for Electronics and Microtechnology*)			X							[113]
Dispensing/sorting robot for small aquatic organisms			X	X						www.lifesciencemethods.com
ARQiv—Automated reporter quantification system in vivo			X	X		X	X			[114]
ScreenCube					X					[66]
Microinjection robot					X					www.lifesciencemethods.com
VAST BioImager (*Union Biometrica*)						X	X	X	X	www.unionbio.com
Imaging robot for small aquatic organisms						X	X	X		www.lifesciencemethods.com
HCS LCI (*Leica*)							X	X	X	www.leica-microsystems.com
Imaging Machine (*Acquifer*)							X	X	X	www.acquifer.de
ImageXpress (*Molecular Devices*)							X	X	X	www.moleculardevices.com
EnSight multimode plate reader (*PerkinElmer*)							X	X	X	www.perkinelmer.com
IN Cell Analyzer (*GE Healthcare*)							X	X	X	www.gehealthcare.com
COPAS Vision (*Union Biometrica*)						X	X	X	X	www.unionbio.com
Micro computed tomography (*Brucker*)								X	X	www.bruker.com
ZebrafishMiner									X	[115]
ZFIQ zebrafish image quantitator									X	[116]
ZFBONE toolset									X	[47]
ImageJ									X	imagej.nih.gov
Image-Pro (*Media Cybernetics*)									X	www.mediacy.com

* all web pages accessed on 22 May 2022.

## Data Availability

Data sharing not applicable.
